# Identifying barriers and facilitators of the implementation of nutrition guidelines in food banks using the Consolidated Framework for Implementation Research

**DOI:** 10.1093/tbm/ibag018

**Published:** 2026-04-13

**Authors:** Violeta Chacón, Isabelli L Costa da Silva, Sarah McKee, Katie Martin, Maisie Campbell, Marlene Schwartz, Caitlin Caspi, Maria Gombi Vaca

**Affiliations:** Rudd Center for Food Policy and Health, University of Connecticut, Hartford, CT, United States; Rudd Center for Food Policy and Health, University of Connecticut, Hartford, CT, United States; Department of Allied Health Sciences, University of Connecticut, Storrs, CT, United States; Food Bank of Western Massachusetts, Chicopee, MA, United States; More Than Food Consulting, LLC, Avon, CT, United States; More Than Food Consulting, LLC, Avon, CT, United States; Rudd Center for Food Policy and Health, University of Connecticut, Hartford, CT, United States; Department of Human Development and Family Sciences, University of Connecticut, Storrs, CT, United States; Rudd Center for Food Policy and Health, University of Connecticut, Hartford, CT, United States; Department of Allied Health Sciences, University of Connecticut, Storrs, CT, United States; Rudd Center for Food Policy and Health, University of Connecticut, Hartford, CT, United States

**Keywords:** food banks, food insecurity, implementation, nutrition guidelines, nutrition policy

## Abstract

**Background:**

The Healthy Eating Research Nutrition Guidelines for the Charitable Food System (HER Guidelines) are nutrition standards to measure and improve the nutritional quality of foods distributed to people experiencing food insecurity.

**Purpose:**

This qualitative study used the Consolidated Framework for Implementation Research (CFIR) to systematically identify the barriers and facilitators to implementing the HER Guidelines in a national sample of US food banks.

**Methods:**

We conducted in-depth interviews among a random sample of food banks that reported implementing the HER Guidelines for at least 1 year. Interviews (12 food banks) were recorded and transcribed. The CFIR was used to create a semi-structured interview guide, coding guide, and thematic analysis. Interrater reliability was assessed by double coding 25% of the transcripts.

**Results:**

Food banks employed different approaches to implementing the HER Guidelines yet often faced similar successes and challenges. Successful HER implementation practices included strategies to prioritize resources, such as focusing efforts on purchased items first and using online tools like WellSCAN to rank foods from USDA (US Department of Agriculture) food programs. Challenges included difficulties evaluating the categories of mixed dishes and grains, evaluating assortments of donated foods, and having enough staff to complete the task of ranking. Food banks expressed the need for more educational resources to train food bank staff members involved in the implementation of the HER Guidelines.

**Conclusion:**

This study identified successful strategies, common challenges, and opportunities to improve the implementation of HER Guidelines to support the availability of healthier food products across the charitable food system.

## Teaser

This study brought to light the significant challenges food banks face, highlighting a critical need for more educational materials. The findings point toward a new path to ensure consistent and effective implementation of the HER Guidelines.

Implications

- **Practice:** To help food banks effectively implement the Healthy Eating Research (HER) Guidelines, interventions should provide resources and training that can be tailored to the unique operational challenges of this setting, acknowledging that structural characteristics such as staff roles and training significantly impact implementation capacity.
- **Policy:** Food banks should design and implement policies that establish nutritional quality of food as a central metric of success, in addition to quantity. This requires increasing resources for educational materials and fostering stronger local and national network collaborations to enable food banks to effectively implement the HER Guidelines.
- **Research:** To advance this work, future research should test interventions such as adaptable training modules to facilitate the implementation of the HER Guidelines. Studies should also explore how scalable interventions are across the diverse operational contexts of food banks and test the effect on the nutritional quality of the foods distributed by food banks.

## Introduction

The charitable food system in the United States is a network of food banks, food pantries, and congregate meal sites that distributes food and household supplies to support households facing food insecurity. Within the charitable food system, food banks are central warehouses that collect and distribute food to partnering agencies, such as food pantries, which are local agencies that directly distribute food to individuals and families. Feeding America is the nation’s largest food relief organization, and it oversees a network of ∼200 food banks, supporting over 60 000 partner agencies [1]. In 2023, over 47 million households in the United States experienced food insecurity at some time during the year [2]. During the same year, the US charitable food system assisted more than 50 million people and distributed more than 5 billion meals [3]. Food insecurity is associated with a higher risk of health conditions, including obesity, diabetes, hypertension, and depression [4–9]. To best support families, many agencies within the charitable food system have begun tracking the nutritional quality of the foods they distribute [10–13].

Food banks across the United States have been tracking nutritional quality using systems like Foods to Encourage, the Choose Healthy Options Program, and Supporting Wellness at Pantries [14]. However, these systems differ in their categorization and ranking criteria. Therefore, in 2020, a national, interdisciplinary expert panel convened to create the Healthy Eating Research Nutrition Guidelines for the Charitable Food System (HER Guidelines) [15]. Importantly, the HER Guidelines were vetted by a second committee of food bankers convened by Feeding America [15, 16]. Since the launch of the HER Guidelines, Feeding America has endorsed the HER Guidelines and provided generous funding and implementation toolkits to encourage food banks to use the HER Guidelines [1]. Similarly, Partnership for Healthier America provided funding and technical assistance to encourage the use of the HER Guidelines [17]. The HER Guidelines are a traffic light rating system with three levels (i.e. ranks): choose often, choose sometimes, and choose rarely. To determine a food rank, the item is placed into one of 11 food categories (i.e. fruits and vegetables, grains, protein, dairy, non-dairy alternatives, beverages, mixed dishes, processed and packaged snacks, desserts, condiments and cooking staples, and miscellaneous items), and then the product’s levels of saturated fat, sodium, and added sugars are compared to specific pre-defined thresholds based on food category. Once the nutrition rank of a food is identified, the information can be added to the item card in food banks’ inventory systems to track the food that enters the food bank.

Classifying foods according to the HER Guidelines is part of a multi-step process of inventory management. There is growing evidence of the value of tracking nutrition information in food banks; however, many food banks face challenges in implementing these processes. One of the important factors is the complexity of food sources and their inventory management. Food banks collect food from various sources. These include the US Department of Agriculture (USDA) commodity food programs, such as The Emergency Food Assistance Program, or the Commodity Supplemental Food Program, or state-level programs [18]; bulk donations from food manufacturers and retailers; and smaller donations from food drives. When food items arrive at food bank warehouses, they need to be sorted and recorded using an inventory management system that tracks item-level information using “item cards.” Inventory item cards typically contain the food item name, description, category (e.g. fresh produce; dairy), source, amount, and storage information. Food banks that track nutrition will also include that information on the item card. The information from the inventory item cards can be used to generate reports and track the nutritional quality of the foods in the inventory. These data are also used to populate online ordering platforms where community partner agencies order food [19].

The HER Guidelines have been implemented, to some extent, in more than 100 food banks and endorsed by Feeding America [20], the largest network of food banks in the United States, and Partnership for a Healthier America, a national organization that partners with food banks across the country to improve access to nutritious foods [21]. However, the level of implementation of HER Guidelines is variable across the charitable food system. In 2023, a landscape assessment conducted by Feeding America and More Than Food Consulting with a sample of 85 food banks across the Feeding America network, found that 59% were using the HER Guidelines to rank food in their inventory, and 44% ranked at least half of the food items in their inventory [22]. This context highlights how the implementation of the HER Guidelines nutrition standards has been challenging in the charitable food system due to limitations in operational capacity and in access to donations and funding to purchase food.

The challenges that food banks face to successfully implement the HER Guidelines have not been formally understood using an implementation science framework. Therefore, this qualitative study, informed by the Consolidated Framework for Implementation Research (CFIR) [23], aimed to systematically identify the barriers and facilitators to implementing nutrition ranking based on the HER Guidelines in a national sample of food banks.

## Methods

### Study design

We used a qualitative study design, based on CFIR, to guide the assessment of HER Guidelines implementation in food banks. This study investigates organizational practices while using an interpretive paradigm, which seeks to understand individuals’ experiences from the perspective of the individuals themselves, while acknowledging that these are subjective, occur within a social, cultural, and historical context, and that the researchers’ backgrounds and values can influence the research data [24]. The Standards for Reporting Qualitative Research checklist [25] was used to ensure the quality of reporting our results.

### Sample selection

**Figure 1:**
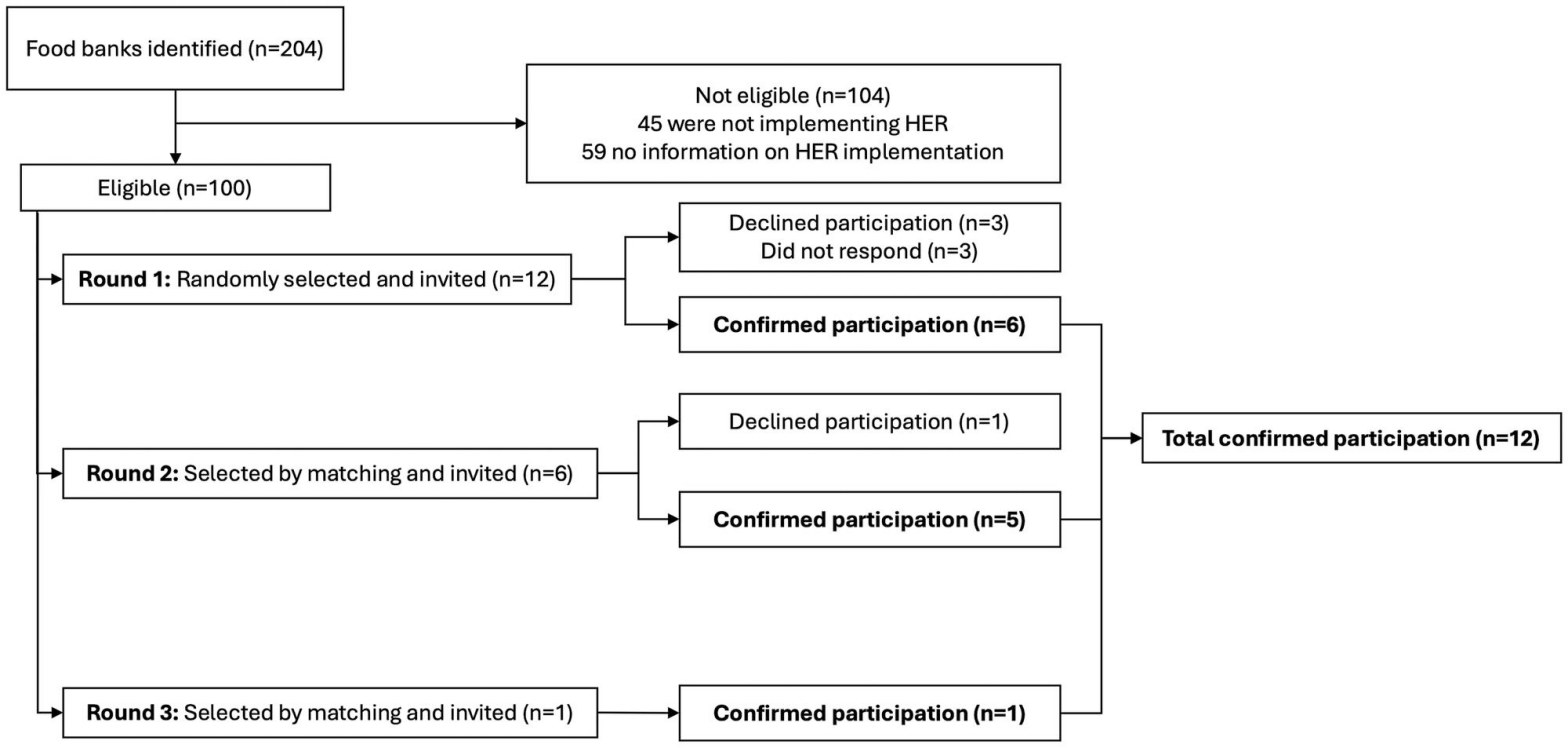
Food bank sample selection.

Participants in this qualitative study were representatives of food banks across the United States. Using secondary data shared by national project partners (Feeding America and the Partnership for Healthier America), a total of 204 active food banks were identified across the United States (Fig. 1). Food banks that had, based on reports from our national partners, implemented HER Guidelines for at least 1 year prior to recruitment were eligible to participate. One hundred food banks that met this criterion were identified by the study team. One hundred and four food banks had either not implemented HER (*n* = 45) or no information on their HER implementation was available (*n* = 59).

The sampling approach aimed to achieve a sample of 12 food banks that reflected the diversity of key food bank characteristics associated with real-world challenges of implementing a nutrition ranking system. The key food bank characteristics shared by national partners considered as the parameters for sample selection were: presence of nutrition staff (yes or no), type of inventory software (i.e. food bank-specific inventory management system: Primarius, Ceres, or other), receipt of grant funds to support HER Guideline implementation (yes or no), and food bank resource and impact level (lower, intermediate, or high). The resource and impact levels were defined based on the food insecurity level in the food bank’s service area, service area size, cost to operate, and resources available. First, using the Stata software splitsample command with the balance() option, the master sample of 100 food banks was randomly divided into eight subsets while ensuring that the distribution of the key food bank characteristics was approximately equal across those subsets. Then, the subset with the most balanced geographic locations of food banks was selected, and these food banks were invited to participate in the study.

Food banks were contacted by email, and food bank staff members who were involved in nutrition ranking (e.g. dietitians, warehouse or procurement staff members) were invited to participate in the interview. In the first round of invitations, 6 of the 12 food banks accepted the invitation to participate in the study. In the second round of invitations, six other food banks were selected to replace those that declined participation, based on matching key characteristics. Of these, five agreed to participate. Another food bank, with matching key characteristics of the one that declined to participate, was invited and agreed to participate. An incentive of $500 was offered to food banks that participated in this study. All interview participants provided written informed consent. This study was approved by the University of Connecticut Institutional Review Board (IRB B2024-0005).

### Data collection

Qualitative data were collected through in-depth interviews designed to foster conversations about the challenges, facilitators, and processes to implement nutrition ranking based on the HER Guidelines. We developed an eight-item semi-structured interview guide using the CFIR [26]. To develop the interview questions, the study team focused on the CFIR domains of innovation characteristics, inner setting, and process. We considered these three domains to be the most relevant barriers or facilitators of the implementation of nutrition ranking using the HER Guidelines. In the context of this study, the HER Guidelines are the innovation we are assessing for complexity and adaptability. Within the inner setting domain, we sought to understand the constructs of food banks’ structural characteristics (e.g. type of inventory management system), work infrastructure (e.g. presence of nutrition staff), and staff access to knowledge and information related to HER Guidelines. Finally, in the process domain, we sought to assess the constructs of planning, engaging, reflecting, and evaluating HER Guidelines implementation.

The interview guide included broad, non-leading questions to allow the participants to share their food bank’s experience with the HER Guidelines. When necessary, we asked probing questions to encourage participants to elaborate on their answers. Interviews were conducted by two interviewers who had experience in qualitative research methods. All interviews were conducted, recorded, and transcribed using Webex and Otter.ai. We conducted 12 interviews with 18 food bank staff members who oversee or are responsible for nutrition ranking foods based on the nutritional content using the HER Guidelines. Participants included food bank staff from teams for procurement (*n* = 4), operations and inventory (*n* = 6), nutrition education, health promotion, and health equity (*n* = 7), and community engagement (*n* = 1). The interviews lasted from 36 to 54 min and were conducted between 6 May and 30 July 2024.

### Researcher characteristics and reflexivity

The team engaged in data collection, analysis, and interpretation consisted of eight researchers who brought various levels of experience working with the charitable food system and qualitative research methods. We recognize that the researchers’ individual backgrounds, experiences, and theoretical understandings shaped the interpretation of the data. To foster transparency and enhance the trustworthiness of our findings, we engaged in reflexivity throughout the research process, which involved regular discussions where we critically examined our potential biases, assumptions, and preconceptions related to the implementation of HER guidelines in the charitable food system.

### Qualitative analysis

We conducted a thematic analysis using both deductive and inductive approaches. First, we generated *a priori* codes based on the three pre-defined CFIR domains (i.e. innovation, process, and inner settings), and the eight subthemes that represent CFIR subdomains (i.e. complexity, adaptability, planning, engaging, reflecting and evaluating, access to knowledge and information, structural characteristics, and implementation climate) (Table 1). We looked for patterns in the data that were not captured by *a priori* codes. One additional subdomain about individuals, based on the CFIR domain of characteristics, emerged. While this domain was not defined *a priori*, it was added as a code to capture the role of individuals’ attitudes, knowledge, and values about the HER Guidelines and its successful implementation. Table 1 details the CFIR domains, subdomains, and definitions in the context of this study.

**Table 1:** Definitions of the Consolidated Framework for Implementation Research subdomains used for this study.

CFIR domain	Subdomains	Definition within the context of this study
Innovation characteristics	Complexity of the HER Guidelines	The challenges and the common mistakes that food banks make when implementing the HER Guidelines.
	Adaptability of the HER Guidelines	The degree to which the HER Guidelines can be adapted, tailored, or refined, to meet the food bank needs.
Process	Planning implementation	The process to identify and record the food item’s information and how these are entered into their inventory system (e.g. creating an item card, systems for naming and assigning identification codes)
	Engagement of staff	The roles and responsibilities of the staff involved with implementing the HER Guidelines.
	Reflecting and evaluating the implementation	The strategies that food banks have in place to monitor and evaluate their HER implementation ranking system.
Inner settings	Access to knowledge and information regarding the HER Guidelines	The resources, guidance, and training available at food banks to implement the HER Guidelines.
	Food bank structural characteristics	The organizational structure of the food banks (e.g. key personnel; software and technology) that are key to implementing the HER Guidelines.
	Implementation climate in food bank	The shared values and policies that guide food banks’ internal practices related to the HER Guidelines.
Individuals’ characteristics	Knowledge and beliefs about the HER Guidelines	Individual attitudes, knowledge, and values placed on the implementation of the HER Guidelines.

Qualitative analysis was conducted using NVivo 14. Two study team members (V.C. and M.G.V) independently coded three (25%) randomly selected transcripts to estimate intercoder reliability. After comparing the independently coded transcripts, minor discrepancies in codes were identified and addressed. The final intercoder reliability was 0.88 on the kappa coefficient for all codes, which indicates strong coding agreement [27].

## Results

Table 2 describes the characteristics of the participant food banks. Most reported the presence of nutrition staff, the use of Ceres or Primarius inventory management systems, and being from the South or West regions of the United States. More than half (58.3%) received funding to implement the HER Guidelines, and most (75%) reported lower or intermediate resource and impact levels. The Supplementary Table presents the characteristics of the food banks that declined participation. Compared to participant food banks, a lower percentage of declining food banks reported having nutrition staff, receiving funding to implement the HER Guidelines, and being from the South or West regions of the United States.

**Table 2:** Characteristics of food bank participants (*n* = 12).

Food bank profile	Frequency, *n* (%)
Presence of nutrition staff	
Yes	9 (75)
No	3 (25)
Inventory management system	
Ceres	5 (41.7)
Primarius	4 (33.3)
Other	3 (25)
Recipient of funding to implement HER Guidelines	
Yes	7 (58.3)
No	5 (41.7)
Resource and impact level	
Lower	4 (33.3)
Intermediate	5 (41.7)
Higher	3 (25)
Geographical region	
Northeast	2 (16.7)
Midwest	2 (16.7)
South	5 (41.6)
West	3 (25)

### Innovation characteristics domain

Within the innovation characteristics domain, participants recognized that ranking inventory foods using the HER Guidelines was a practice that had been adopted somewhat recently in their organizations. Within this theme, participants described the challenges that contribute to the complexity of adopting the HER Guidelines and how they wish to adapt them to fit their context and needs.

#### Subdomain complexity of the HER Guidelines

Participants mentioned several challenges while implementing the HER Guidelines. The most common challenge was ranking the category of assorted food donations. This occurs when a mix of different types of products (that may contain a variety of foods with different levels of saturated fat, sodium, and added sugar) are donated together due to a food drive (e.g. a mix of canned and packaged goods) or a retailer donation (e.g. assorted frozen meats; assorted bakery items). Ranking these foods is challenging because they require more resources (e.g. staff, time, and space) to sort, categorize, and rank them compared to bulk donations (i.e. all items are the same product) that are already organized or palletized when they enter the warehouse. Some participants said that, in the past, they had made efforts to rank these without success and because it was so time-consuming, many decided not to rank them going forward. Food banks usually leave these without a rank or label them as “Assorted-Not ranked” in their inventory system.

There’s a huge portion of what we receive that comes in, with multiple items on a pallet, it’s assorted foods. And to go through those you know pallet by pallet, there’s just not the manpower. (Director of Operations)

Another prominent challenge identified by participants was navigating the ambiguity in categorizing and ranking certain foods. For instance, common dilemmas emerged around whether items such as breakfast cereals and granola bars should be ranked as grains or processed snacks. Similar confusion arose regarding the ranking of foods that contain ingredients from more than one food group. One participant, e.g. debated the categorization of mashed potatoes as a mixed dish versus a vegetable.

You know, something like mashed potatoes. Like, is that a mixed dish? Is that a vegetable? … we had things like pizza rolls, we struggled with where to put it? … Is that a snack or is that a meal? Is it the right place? (Inventory Manager)

Another related issue is that the staff in charge of implementing the HER Guidelines sometimes had specific ideas about the healthfulness of certain foods. For example, some participants questioned whether canned meats like tuna in water merited a “choose often” ranking. Other participants noted discrepancies between other nutrition quality tracking systems used in food banks, such as “Foods to Encourage” (i.e. Feeding America’s internal system of designating some food categories as healthy for measurements of the food distributed by the network) compared to the HER Guidelines.

I think cereal is a prime example around all the dialogue that takes place. From a Foods to Encourage perspective, it’s 100%, [of cereals are] nutritious. From an HER perspective, it all depends on where it falls within three categories, sugar, fat, and sodium. (Inventory Manager)

Some participants said that it is important for their organizations to source items that are healthy. Therefore, they use the HER Guidelines to identify products that are ranked as “choose often” prior to purchasing them. However, food banks also noted that this could be difficult due to the lack of nutrition information on vendors’ websites or changing nutrition profiles due to product reformulation. This is challenging for the food banks that use the same item card for any version of a food (e.g. just one item card for green beans, even though HER rank may vary across brands). In that case, food items that have already been entered into their inventory system with an item card and HER rank need to be updated to a new item card for the same product and a different HER rank.

I’ll be honest with you; there’s a lot of maintenance to keep it up to date. Again, the can of green beans, the same can of green beans came in, it was yellow, but suddenly is green… (Director of Operations)

#### Subdomain adaptability of the HER Guidelines

During the interviews, participants talked about aspects of the innovation that could be changed, tailored, or refined to fit their organization’s nutrition standards. Participants talked about changes in food banks’ infrastructure and equipment, such as the ability to update their inventory software system toward adapting the HER Guidelines into a more automated ranking process. One participant suggested using scanners to capture item information, which could then be automatically categorized and ranked.

If there were a scanner that could tell you what the ranking was, that I think that would be helpful… the first thing that comes to mind would be some sort of scanner that kind of eliminates the human component from it. And it’ll just tell you what that is, right away without you having to make that decision yourself with the chart. (Director of Product Sourcing)

### Process domain

Participants talked at length about the activities and strategies they use to implement the HER Guidelines in their food bank. They identified the staff roles, outlined the specific steps within their operations, and discussed how their food bank evaluates the success of the implementation.

#### Subdomain planning implementation

In terms of the specific steps that food banks take to implement the HER Guidelines, participants talked about how they identify, rank, and record the items that come into their warehouse. The item identification process was similar across food banks. They usually create item cards for products when they are purchased or received at the warehouse. The information recorded in item cards by food banks varied, but usually included name, identification number, weight, food category, price, and origin (e.g. purchased, donation, USDA). Some food banks shared that they record nutrition information and take pictures of the front and back of products to store this information for future reference.

Participants also talked about the ways that they strategically focus their resources in different areas to implement the HER Guidelines according to their operational capacity, prioritizing ranking purchased or sorted food at various stages of operations and deprioritizing ranking mixed donations. One approach is to integrate the HER Guidelines in the procurement process. By ranking foods before acquisition, food banks can make informed purchasing decisions to obtain healthier items.

…the only thing that we’re ranking is purchase products… And one of the reasons why we only do purchase is because of the challenges in ranking donated goods. (Director of Product Sourcing)

Another approach to integrate the Guidelines into their operations is to rank foods when they are received at the warehouse. Food banks that took this approach invested in training their warehouse staff to rank incoming food items as they arrived. However, to address the challenges of ranking incoming donated items, some food banks prioritize the ranking of items that are easier to categorize, like fresh or canned vegetables, and items that arrive in large quantities. Some participants expressed a desire to see changes on factors external to food banks. For instance, some participants wished that donors would separate their donated products into boxes with the same HER food category to help food banks’ staff rank items more quickly.

We currently only rank case-lotted donated products. So, if it’s in a case, we will rank those. But if there’s a mixed box of donated product, obviously we don’t have the time, capacity or manpower to take each individual item and put it through the ranking system. (Food Sourcing Support Coordinator)

And then for donated, pallet sized items, our team ranks them at the time of receiving them. So they’ll create the item card and put the ranking into our inventory system. And we have a large poster that has the guidelines on it. (Health and Nutrition Program Manager)

Finally, some food banks opted to implement the HER Guidelines at a later stage, specifically during the inventory reporting phase. This method typically involved the food bank’s dietitian or nutrition staff reviewing the item card information in the inventory system and systematically ranking items based on the information available. In this case, the HER Guidelines were applied retrospectively to rank foods after they had been received and inventoried.

#### Subdomain engagement of staff

In terms of the roles and responsibilities for implementation of the HER Guidelines, participant food banks take different approaches. Some food banks rely on just one staff member to do all the ranking; this minimizes the mistakes and the need for ongoing training. Other food banks include staff from the warehouse, inventory, and procurement departments involved in the ranking process.

Participants also mentioned the issue of high staff turnover as one of the challenges related to engagement that impacts the implementation of the HER Guidelines at their food banks. Many participants noted that a high percentage of employees leave, and new staff are hired within short periods of time. This constant influx of new staff requires frequent training and increases the risk of ranking mistakes, making it more difficult to maintain a standardized and accurate ranking system.

We’ve gone through some staff changes in the past year. And so, there were people that were versed in HER guidelines. And then now it’s just me and my team. (Health Promotion Specialist)

#### Subdomain reflecting and evaluating the implementation

The participants did not report a consistent strategy to monitor the HER Guidelines implementation. Some food banks mentioned that they are focused on the completeness (e.g. the proportion of items ranked) while others said that they are primarily interested in their accuracy (e.g. reviewing ranks of foods before making the information available to partner agencies). Some participants also mentioned that they pull reports from their inventory software that identify the proportion of pounds by food category (e.g. grains; dairy), rank (e.g. rank vs unranked), and origin (e.g. USDA or donation) to discuss with their team and inform grant proposals or program evaluations.

### Inner settings domain

Within the inner settings of food banks, participants talked about the resources that they use to access knowledge and information to implement HER Guidelines; the key staff members who are involved in HER Guidelines implementation; the usefulness of the software inventory system to implement HER Guidelines; and the internal nutrition policies and practices related to the HER Guidelines.

#### Subdomain access to knowledge and information regarding the HER Guidelines

The main resource that participants use to rank items is the one-page chart “Nutrition Guidelines for Ranking Charitable Food,” which is part of the original, full report [15]. The chart is a user-friendly summary of the key nutrient thresholds for each food category to determine whether a food is choose often, sometimes or rarely. Many food banks said that they have a printed version of the chart on hand when they are ranking food items. Another resource that most participants mentioned is the WellSCAN website [28], which is an online tool that allows food banks to identify and rank specific food items. Some food banks were aware of this tool and have been using it to rank and train new staff and volunteers.

We had been using the WellSCAN app, which was a useful tool for us, and especially for those receiving team folks who may not have as much nutrition knowledge to utilize that tool to help capture the nutrition ranking. (Nutrition Education Supervisor)

However, some participants did not know about this tool and, when prompted about it, they showed interest in learning how to use it. Some food banks highlighted the use of web-based tools (e.g. Google Search, https://www.google.com; Nourish Calculator https://nourish.us.org) to find nutritional information of products and help with their nutrition ranking efforts.

In addition, participants said that they wish to have more adaptable educational materials to train new staff members or conduct regular educational sessions to refresh the HER Guidelines with the existing staff.

I always love … those templates that you can make those edits, and you can add your food bank logo, like that’s always really helpful. So … having those different resources that again, can be sent to different audiences. (Nutrition Education Supervisor)

Some participants also mentioned their wish to connect with other food banks to learn from their experiences with the HER Guidelines.

Just gathering information from other people is where I’ve learned what I’ve learned and found the motivation to keep going with it. (Purchasing and Agricultural Food Resources Manager)

#### Subdomain food bank structural characteristics

Within the organizational structure, food banks mentioned two types of expertise on staff were key to implementing the HER Guidelines. First, they mentioned that having a nutrition staff on the team, such as a Registered Dietitian or a dietetic intern, was instrumental in setting up the process and training the other staff to rank foods using the Guidelines.

But then I also delegate some of that work to the dietetic interns helping us out, we get dietetic interns every month. We also have volunteers, so we call them community champions who are specifically housed with nutrition. So we use some of their help. (Health Promotion Specialist)

The second key staff member mentioned was a person with Information Technology (IT) expertise, who can facilitate the inventory software setup for ranking, monitoring, and reporting the use of HER Guidelines in their organization. Participants largely said that the inventory software systems they use, independently of the brand, were generally straightforward and easy to use for ranking foods. While initial setup and familiarity with the system required time and practice, respondents indicated that the process of recording each item’s rank itself was efficient after the system was configured. Participants mentioned that they often connect and collaborate with other food banks in the network to discuss IT issues. Several mentioned challenges when it came to pulling reports that detail item cards and their HER ranks from these systems and noted the need for a key IT or a software-knowledgeable person to help monitor and report on inventory ranking.

I’d say the biggest challenge we have right now with the whole system is the reports are hard to build… we have to figure out how to build those reports because you never know what could happen in the world, you know, all of a sudden you don’t have your data person. (Operations Manager)

#### Subdomain implementation climate in food bank

Reflecting how the HER Guidelines are related to shared values and policies, some participants said that they have formal nutrition policies and plans to integrate the Guidelines into their internal operations. For example, participants mentioned setting goals for procuring a minimum of items ranked as “choose often,” and incorporating the rank into the online shopping platform used by their partner organizations. However, some participants are still in the process of developing their nutrition policies with the aim of using the HER Guidelines as a framework. Food bank nutrition policies mentioned by participants often included goals to increase the proportion of foods that are ranked “choose often” or “choose sometimes,” standards for procurement, educational initiatives for partner agencies, periodic education for the staff that implements the HER Guidelines and holding regular meetings to discuss progress.

I lead our action team. And so that started in March of 2023. And so that was a space where we created goals together around the implementation of the HER nutrition guidelines, had conversations with individuals to make sure that any implementation steps are feasible or realistic. (Nutrition Education Supervisor)

Within the food banks’ internal practices, participants described efforts to communicate about the HER Guidelines work with their partners and donors. In some food banks, the HER rank for foods is available in the ordering systems used by agency partners to obtain foods from food banks. Many participants mentioned that they educate their partners about the HER Guidelines and what the recommended nutrient levels mean when selecting foods. Materials and tools used in these educational programs included videos, handouts, periodic meetings/conferences, newsletters, and site visits. However, some food banks expressed a desire to engage more proactively with donors, educating them about the nutritional quality of foods sought within the charitable food system and advocating for healthier donations. Others suggested that donors could organize donations into pre-categorized boxes, thereby streamlining internal processes for sorting and ranking inventory.

Regarding funding, only a few food banks mentioned receiving funds to support specifically the implementation of HER guidelines. In these food banks, participants noted that the funding was usually used for acquiring and setting up software to implement the Guidelines, for educational materials, and to implement programs to increase their partners’ capacity to adopt the HER Guidelines.

### Individuals’ characteristics domain

#### Subdomain knowledge and beliefs about the HER Guidelines

Throughout the interviews, participants reflected and expressed beliefs and feelings about their work with the HER Guidelines. Many food banks expressed the hope that the HER Guidelines could help them offer more healthy options to the communities they serve. They also hope that more food banks across the country will start implementing the Guidelines. Participants were passionate about their work at the food banks and wished they had more resources to prioritize nutrition when procuring foods.

And they’re [food bank colleagues] pretty passionate about ranking. And they know that I’m passionate about ranking. (Purchasing and Agriculture Food Sourcing Coordinator)

However, some participants also expressed frustration at the number of foods that they are not able to rank, especially assorted donations. They also said that they get overwhelmed by all the work that the food bank operations involve, which sometimes prevents them from dedicating more time to the healthfulness of the products. Some participants said that they would like to have donors that are more conscious of the healthfulness of foods that they donate.

## Discussion

This qualitative study assessed the barriers and facilitators to implementing the HER Guidelines using the CFIR in a national sample of food banks. We found that food banks have different approaches to implementing the HER Guidelines, but many face common challenges. These challenges include ranking food categories such as mixed meals and grains or ranking broad assortments of food that arrive unsorted. Structural characteristics of food banks, including staff make-up and training, also impact their capacity to implement the HER Guidelines. Results also revealed that food banks need more adaptable educational resources to train food bank staff members involved in the implementation of the HER Guidelines, especially due to a common challenge of staff turnover. Successful HER Guideline implementation practices also emerged, including creative strategies that food banks use to allocate resources to implement the HER Guidelines within their unique operational resources. Strong sentiments of hope for the future and passion for food bank work illustrate the deep commitment of people working in food banks to provide healthier food choices for their communities.

Participants described a range of strategies for implementing the HER Guidelines. According to their capacity and goals, food banks focus their efforts on ranking items that come from a specific source or at a certain phase of the process. In this study, we found that most food banks start their ranking process with purchased items. This is consistent with a recent qualitative study among a sample of food bank executives across the United States, which found that, to advance nutrition-focused initiatives, food banks prioritize their resources on strategies that intersect with food sourcing (i.e. supporting healthier food inventory), handling, and operations [29]. While this allows food banks to rely on their own strengths, it also reveals opportunities for improving the process of implementing the HER Guidelines. For example, food banks that are planning to implement the HER Guidelines may start the implementation by ranking items for which nutritional information is available ahead of time. This is the case of purchased items, or the foods from the USDA food programs, which HER ranks have been previously assessed [30] and are available through the WellSCAN online tool [28].

The analysis of the interviews revealed a critical need for enhanced educational materials and resources to support food bank staff in effectively implementing the HER Guidelines. Participants expressed their desire for adaptable educational materials that could be tailored to their specific operational needs and available resources. For example, training opportunities and friendly materials for new staff joining food banks who are learning about the HER Guidelines for the first time. This is consistent with the results from a mixed methods study among food pantry staff in Boston, that found that one of the main facilitators to improve healthier food choices was a customizable and accessible training for staff members [31]. This is particularly important due to the frequent staff turnover and shortage reported by many food banks [31, 32].

Another opportunity highlighted by our results is the need to foster stronger network connections and collaboration across food banks to encourage knowledge sharing and inform best practices and nutrition policy development. As part of grant funding opportunities, Feeding America and Partnership for a Healthier America hosted peer learning sessions for funded food banks. These calls served as a place to share new resources, innovative practices and collectively brainstorm around common challenges [17]. This need has also been identified in past research that described best practices to advance nutrition policies across food banks in the United States [29]. Addressing these educational and resource needs could contribute to a more consistent adoption and implementation of the HER Guidelines, ultimately enhancing the quality of the foods provided to communities in need.

Our findings suggest that reporting and evaluating the HER Guidelines implementation within food banks represent an area with considerable potential for growth. A robust reporting strategy is critical to enable food banks to set clear goals and effectively communicate objectives to board members and donors [16]. In addition, developing a plan for evaluation with specific details about how and when to audit the implementation of HER Guidelines is also a best practice recommendation to ensure the quality and healthfulness of the distributed foods [33, 34]. For instance, comprehensive reports could help identify food groups or food donors that have large percentages of “choose rarely” items which can lead to more intentional procurement decisions. However, participants frequently reported challenges in generating these reports from their existing inventory systems. Moreover, only a few participants reported having a clear and explicit strategy for evaluating and monitoring their HER Guidelines implementation. Strengthening reporting capabilities and developing explicit strategies (e.g. providing report templates or creating consistent reporting standard units) could enhance food banks’ capacity to track their progress, identify areas for improvement, and reinforce their commitment to their collaborators.

Our study also revealed discussions about food banks’ desire for greater influence over the nutritional quality of donated foods. While many participants said they educate their partners about the HER Guidelines, some would engage more proactively with donors about the desired nutritional quality of the donated foods. A recent scoping review describing policy approaches to nutrition among food banks found that the unpredictability of donated foods and reliance on donations are important barriers to providing healthy foods to the community [33, 34]. According to the 2024 Feeding America Annual Report, almost 58% of the meals distributed by the Feeding America network were food rescued (i.e. donated) by working with retailers, manufacturers, and growers [35]. Our results align with other studies that have also documented food bank efforts to request more healthful foods from donors or even refuse unhealthful donations in accordance with their nutrition policies [25]. In our study, food banks expressed a desire to increase awareness of the HER Guidelines to advocate for healthier donations. However, the level of awareness of the HER Guidelines among donors remains unknown. These findings underscore the critical role of the external factors, particularly donor engagement and education, in shaping the implementation of the HER Guidelines within the charitable food system, and the need for future research on how stakeholders’ awareness and policy decisions are in alignment with the guidelines.

Finally, individuals’ motivation to implement HER Guidelines was also stressed across all interviews. Participants are passionate about their work at the food banks and wish they had more resources to prioritize nutrition when procuring foods. This highlights the importance of supporting the implementation of HER Guidelines, to develop resources to overcome barriers to their adoption, and to ensure that food bank nutrition policies are backed by nutrition standards. These efforts can guide food banks with the aim of benefiting the community they serve by distributing more nutritious and healthier foods.

### Strengths and limitations

This qualitative study has several strengths. First, this study offers insights into the facilitators, opportunities, and capabilities of food banks to implement the HER Guidelines. This is important given that more than 100 food banks across the country have already started implementing the HER Guidelines [36]. Second, this study includes a diverse sample of food banks, which maximizes the external validity of these results and ensures the inclusion of food banks that serve various and distinct communities across the United States. Third, the qualitative nature of the study allowed us to make an in-depth exploration of the barriers and challenges that food banks experience while implementing the HER Guidelines. Fourth, the qualitative data also allowed us to understand a range of approaches that food banks use to capitalize on the resources that they have. Finally, this study also has the potential to identify and guide the development of resources to improve the implementation of HER Guidelines.

This study also has some limitations. The interpretation of qualitative data is inherently subjective and might potentially lead to varying conclusions. In addition, interviewers can also introduce bias by influencing interviewee’s responses, which can also shape conclusions of the study. However, qualitative data from in-depth interviews with food bank staff members are important to understand the individual perspectives and experiences. Another potential limitation of our study is that participants may have felt pressure to provide responses that were favorable to the HER Guidelines or their organization’s operational procedures, potentially limiting the expression of critical perspectives. Another limitation is that, despite the efforts to include a diverse group of staff, our sampling may not have captured all the individuals and roles critical to the everyday implementation of the HER Guidelines.

As more food banks across the nation adopt the HER Guidelines, these nutritional standards have the potential to shift the focus from the traditional metrics (e.g. pounds of food) to nutritional quality and become a central measure of success of food banks’ impact, shaping a healthier food system across the communities they serve. Our study also identified the challenges food banks face while implementing the HER Guidelines, which highlight the critical need for more adaptable and user-friendly educational materials to ensure consistent and effective implementation. Food banks in this study were balancing challenges related to the complexity of ranking food with a sense of hope and passion for providing healthy food to their communities.

## Supplementary Material

ibag018_Supplementary_Data

## Data Availability

Data from this study are not available in a public archive and cannot be shared according to institutional IRB standards.

## References

[1] Feeding America. https://www.feedingamerica.org/ (10 July 2025, date last accessed).

[2] Rabbitt MP , Reed-Jones M, Hales LJ, Burke MP. United States Department of Agriculture. Economic Research Service. *Household Food Security in the United States in 2023*. Washington, DC: Economic Research Service, U.S. Department of Agriculture, 2024. https://handle.nal.usda.gov/10113/8583175 (15 April 2025, date last accessed).

[3] Feeding America. *Charitable Food Assistance Participation | Feeding America*. https://www.feedingamerica.org/research/charitable-food-assistance-participation (14 April 2025, date last accessed).

[4] Caspi CE , Davey C, Barsness CB et al Applying the Healthy Eating Index-2015 in a sample of choice-based Minnesota food pantries to test associations between food pantry inventory, client food selection, and client diet. J Acad Nutr Diet 2021;121:2242–50. 34103273 10.1016/j.jand.2021.05.007 PMC8530893

[5] Simmet A , Depa J, Tinnemann P et al The dietary quality of food pantry users: a systematic review of existing literature. J Acad Nutr Diet 2017;117:563–76. 27727100 10.1016/j.jand.2016.08.014

[6] Gregory CA , Coleman-Jensen A. *Food Insecurity, Chronic Disease, and Health Among Working-Age Adults*. Report No.: 235. US Department of Agriculture, Economic Research Service, 2017. https://ageconsearch.umn.edu/record/261813/ (19 September 2024, date last accessed).

[7] Leung CW , Kullgren JT, Malani PN et al Food insecurity is associated with multiple chronic conditions and physical health status among older US adults. Prev Med Rep 2020;20:101211. 32983850 10.1016/j.pmedr.2020.101211 PMC7502278

[8] Laraia BA. Food insecurity and chronic disease. Adv Nutr Bethesda Md 2013;4:203–12. 10.3945/an.112.003277 PMC3649100 23493536

[9] Gundersen C , Ziliak JP. Food insecurity and health outcomes. Health Aff Proj Hope 2015;34:1830–9. 10.1377/hlthaff.2015.0645 26526240

[10] MAZON-Report-TippingPoint.pdf. https://mazon.org/wp-content/uploads/MAZON-Report-TippingPoint.pdf (15 April 2025, date last accessed).

[11] Caspi CE , Grannon KY, Wang Q et al Refining and implementing the Food Assortment Scoring Tool (FAST) in food pantries. Public Health Nutr 2018;21:2548–57. 29808784 10.1017/S1368980018001362 PMC6729128

[12] Martin KS , Wolff M, Callahan K et al Supporting wellness at pantries: development of a nutrition stoplight system for food banks and food pantries. J Acad Nutr Diet 2019;119:553–9. 29728328 10.1016/j.jand.2018.03.003

[13] Wetherill MS , White KC, Seligman H. Charitable food as prevention: food bank leadership perspectives on food banks as agents in population health. Community Dev (Columb) 2019;50:92–107. 31057344 10.1080/15575330.2019.1570961 PMC6497174

[14] Fledman M , Schwartz MB. A tipping point: leveraging opportunities to improve the nutritional quality of food bank inventory. MAZON: A Jewish Response to Hunge, 2018, 1–20. https://mazon.org/wp-content/uploads/MAZON-Report-TippingPoint.pdf (10 July 2025, date last accessed).

[15] Schwartz M , Levi R, Lott M et al Healthy Eating Research Nutrition Guidelines for the Charitable Food System. Durham, NC: Healthy Eating Research, 2020. https://healthyeatingresearch.org/wp-content/uploads/2020/02/her-food-bank_FINAL.pdf (1 April 2024, date last accessed).

[16] Levi R , Schwartz M, Campbell E et al Nutrition standards for the charitable food system: challenges and opportunities. BMC Public Health 2022;22:495. 35287656 10.1186/s12889-022-12906-6 PMC8919136

[17] Partnership for a Healthier America. *Healthy Hunger Relief*. https://www.ahealthieramerica.org/healthy-hunger-relief-22#resource_grid-301 (11 July 2025, date last accessed).

[18] Feeding America. *How Food Banks Get Their Food | Feeding America*. https://www.feedingamerica.org/hunger-blog/how-food-banks-and-food-pantries-get-their-food (15 April 2025, date last accessed).

[19] Lasavath V. *What’s the Difference Between a Food Pantry and a Food Bank?* Los Angeles Regional Food Bank, 2021. https://www.lafoodbank.org/stories/difference-between-food-bank-and-food-pantry/ (15 April 2025, date last accessed).

[20] Feeding America. *Nutrition in Food Banking Toolkit*. Chicaco, IL: Feeding America, 2021.

[21] Partnership for a Healthier America. *2023 Progress Report*. https://www.ahealthieramerica.org/progress-reports/2023 (15 April 2025, date last accessed).

[22] Feeding America. *Feeding America Survey about HER Nutrition Guidelines Implementation*. Feeding America Network Activity Report (NAR). Chicago, IL: Feeding America, 2023, 1–3.

[23] Damschroder LJ , Reardon CM, Widerquist MAO et al The updated Consolidated Framework for Implementation Research based on user feedback. Implement Sci 2022;17:75. 36309746 10.1186/s13012-022-01245-0 PMC9617234

[24] Prasad P. Crafting Qualitative Research: Working in the Postpositivist Traditions. New York: Routledge, 2015, 352 p.

[25] O’Brien BC , Harris IB, Beckman TJ et al Standards for reporting qualitative research: a synthesis of recommendations. Acad Med 2014;89:1245–51. 24979285 10.1097/ACM.0000000000000388

[26] CFIR Research Team Center for Clinical Management Research. *Consolidated Framework for Implementation Research (CFIR)*. 2023. https://cfirguide.org/ (15 May 2025, date last accessed).

[27] Landis JR , Koch GG. The measurement of observer agreement for categorical data. Biometrics 1977;33:159–74. 843571

[28] *WellSCAN - Nutrition Ranking Technology for Food Banks and Food Pantries*. https://wellscan.io/ (23 April 2025, date last accessed).

[29] Wetherill MS , White KC, Seligman HK. Nutrition-focused food banking in the United States: a qualitative study of healthy food distribution initiatives. J Acad Nutr Diet 2019;119:1653–65. 31262694 10.1016/j.jand.2019.04.023 PMC6765436

[30] Gombi-Vaca MF , Schwartz MB. Evaluation of US department of agriculture foods programs for households using nutrition guidelines for the charitable food system. J Acad Nutr Diet 2023;123:1061–74. 36841356 10.1016/j.jand.2023.02.012

[31] Jia J , Burgun R, Reilly A et al A food bank program to help food pantries improve healthy food choices: mixed methods evaluation of the Greater Boston Food Bank’s Healthy Pantry Program. BMC Public Health. 2023;23:355. 10.1186/s12889-023-15243-4 36797729 PMC9936683

[32] Lohnes J , Wilson B. Bailing out the food banks? Hunger relief, food waste, and crisis in Central Appalachia. Environ Plan Econ Space 2018;50:350–69. 10.1177/0308518X17742154

[33] U.S. Centers for Disease Control and Prevention. *Nutrition*. 2024. Strategies for Food Service and Nutrition Guidelines. https://www.cdc.gov/nutrition/php/public-health-strategy/food-service-and-nutrition-guidelines.html (4 June 2025, date last accessed).

[34] *Implementing and Evaluating Nutrition Policies and Standards in Food Pantries FINAL UPDATED.pdf*. https://nopren.ucsf.edu/sites/g/files/tkssra5936/f/Implementing%20and%20Evaluating%20Nutrition%20Policies%20and%20Standards%20in%20Food%20Pantries%20FINAL%20UPDATED.pdf (4 June 2025, date last accessed).

[35] Feeding America. *Annual Report 2024*. https://www.feedingamerica.org/sites/default/files/2024-12/FA_24AnnRep_d5_ONLINE.pdf (3 August 2025, date last accessed).

[36] Costanzo C. *Food Bank News* 2023. *100+ Food Banks are Using HER Nutrition Guidelines*. https://foodbanknews.org/100-food-banks-are-using-her-nutrition-guidelines/ (23 April 2025, date last accessed).

