# Supplementary material for: Identifying barriers and facilitators of the implementation of nutrition guidelines in food banks using the Consolidated Framework for Implementation Research
Source: Transl Behav Med. 2026 Apr 13;16(1):ibag018. doi: 10.1093/tbm/ibag018 (PMC13076927; doi:10.1093/tbm/ibag018)
Supplement: ibag018_Supplementary_Data [file ibag018_supplementary_data.docx]

**Supplemental table: Characteristics of food banks that declined study participation (n=7).**

| **Food Bank Profile** | **Frequency, *n (%)*** |
| --- | --- |
| *Presence of nutrition staff* | |
| Yes | 4 (57.1) |
| No | 3 (42.9) |
| *Inventory management system* | |
| Ceres | 5 (71.4) |
| Primarius | 2 (28.6) |
| Other | 0 (0) |
| *Recipient of funding to implement HER Guidelines* | |
| Yes | 3 (42.9) |
| No | 4 (57.1) |
| *Resource and impact level* | |
| Lower | 4 (57.1) |
| Intermediate | 1 (14.3) |
| Higher | 2 (28.6) |
| *Geographical region* | |
| Northeast | 1 (14.3) |
| Midwest | 3 (42.8) |
| South | 2 (28.6) |
| West | 1 (14.3) |
